# Supplementary figures and images for: Inclusion of maintenance energy improves the intracellular flux predictions of CHO
Source: PLoS Comput Biol. 2021 Jun 11;17(6):e1009022. doi: 10.1371/journal.pcbi.1009022 (PMC8221792; doi:10.1371/journal.pcbi.1009022)

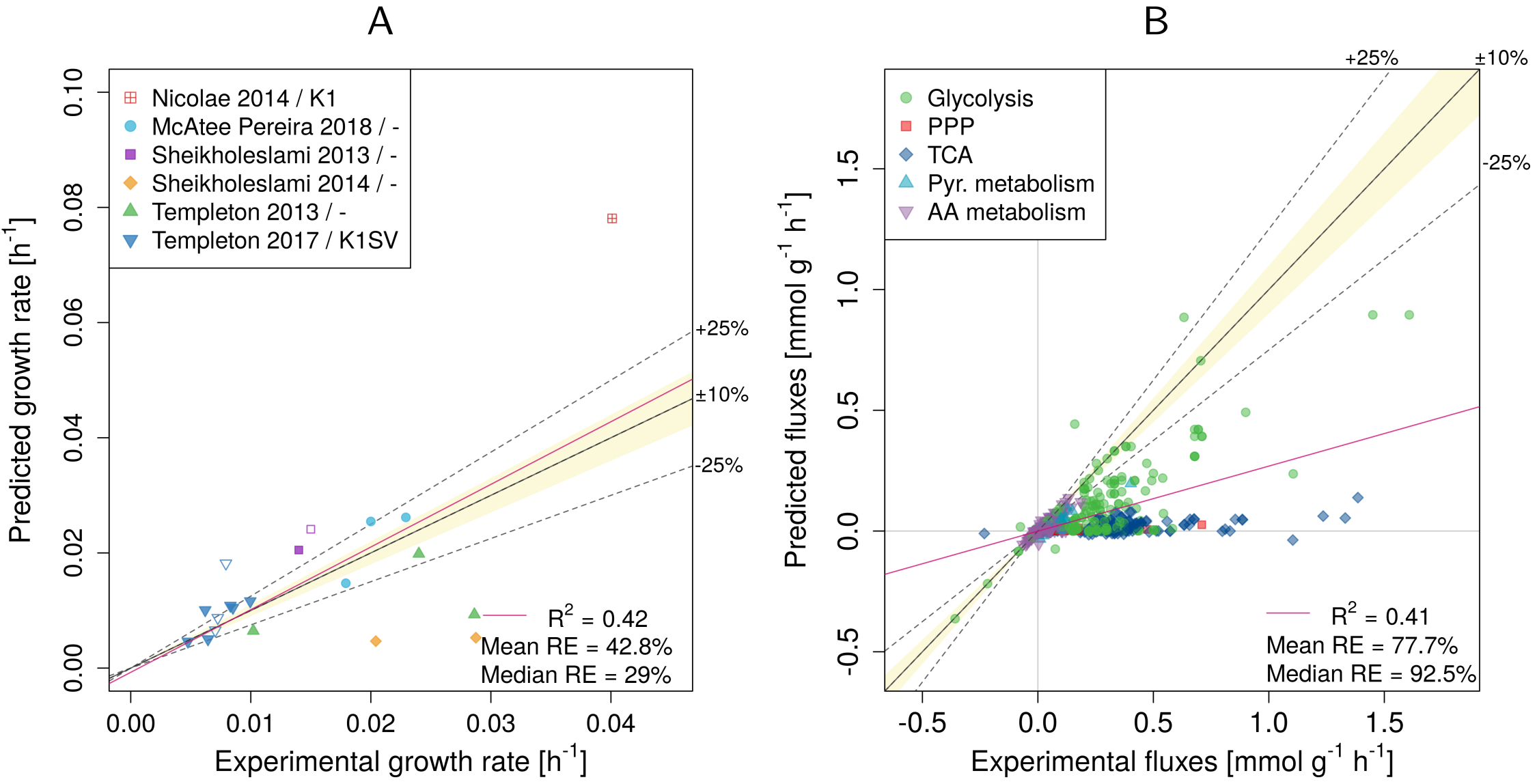

Supplement: S1 Fig — Data is shown for biomass equation R_biomass_cho_producing as the objective function. RE—relative error. The legend in panel (A) indicates the publication and the used CHO cell line (if the information was available). Empty symbols indicate non-producers. (TIF) [file pcbi.1009022.s003.tif]

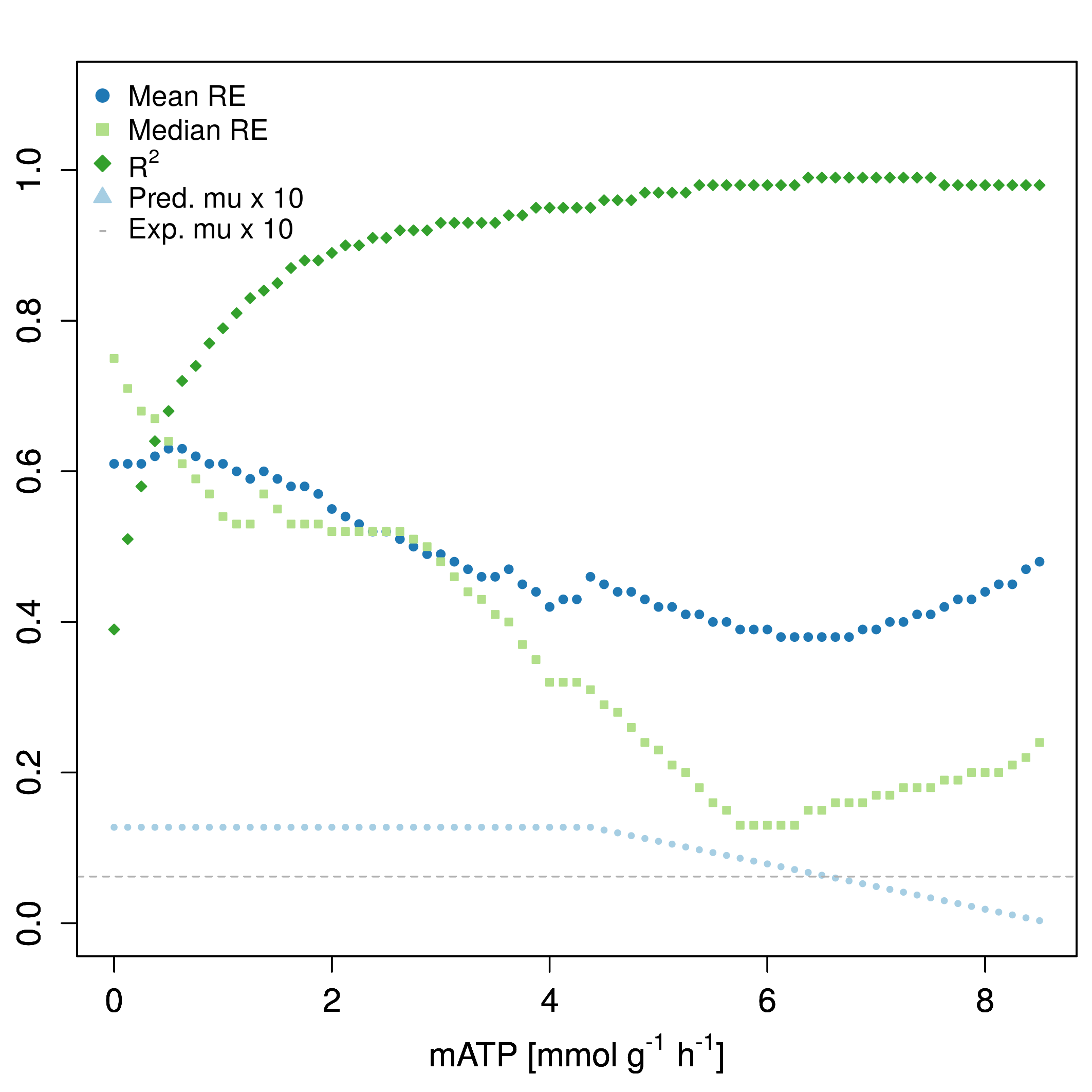

Supplement: S2 Fig — mATP was gradually increased and the agreement between experimental and predicted fluxes was evaluated at each step. The mATP value that lead to the smallest median relative error of the fluxes was chosen as the optimal value. Data is shown for the dataset SV-M3 from Templeton 2017 [22] for biomass equation R_biomass_cho. (TIF) [file pcbi.1009022.s004.tif]

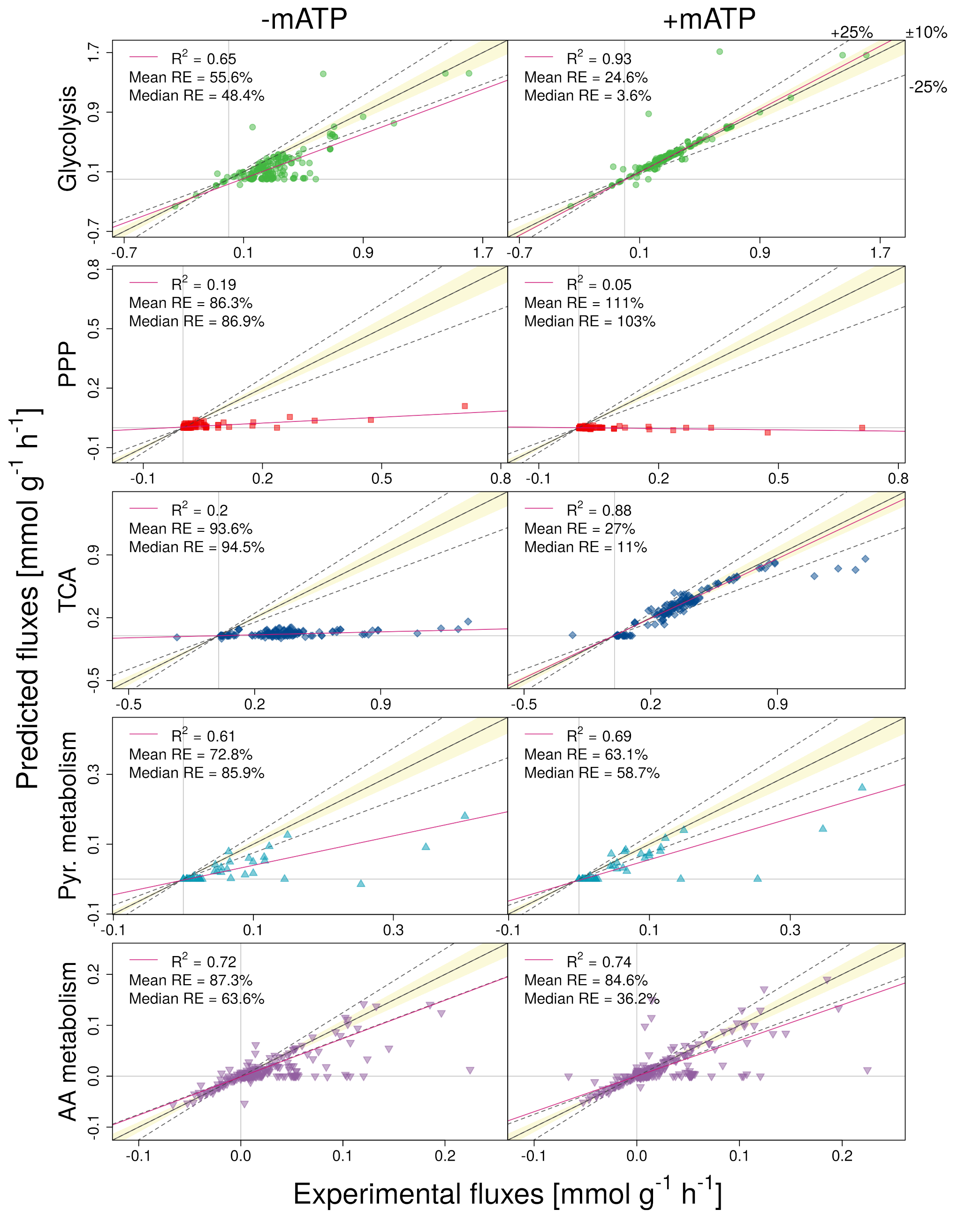

Supplement: S3 Fig — Results are shown for R_biomass_cho as the objective function. RE—relative error. Several outliers can be observed, which in most cases belong to a specific dataset. For example in glycolysis +mATP, the two most overestimated points belong to the “K1” dataset. In TCA +mATP, four underestimated points again belong to “K1” dataset, one to “early” dataset. In pyruvate metabolism +mATP, two underestimated points belong to “K1”, two to “early” datasets. In AA metabolism, the outliers belong to various datasets and sub-pathways. (TIF) [file pcbi.1009022.s005.tif]

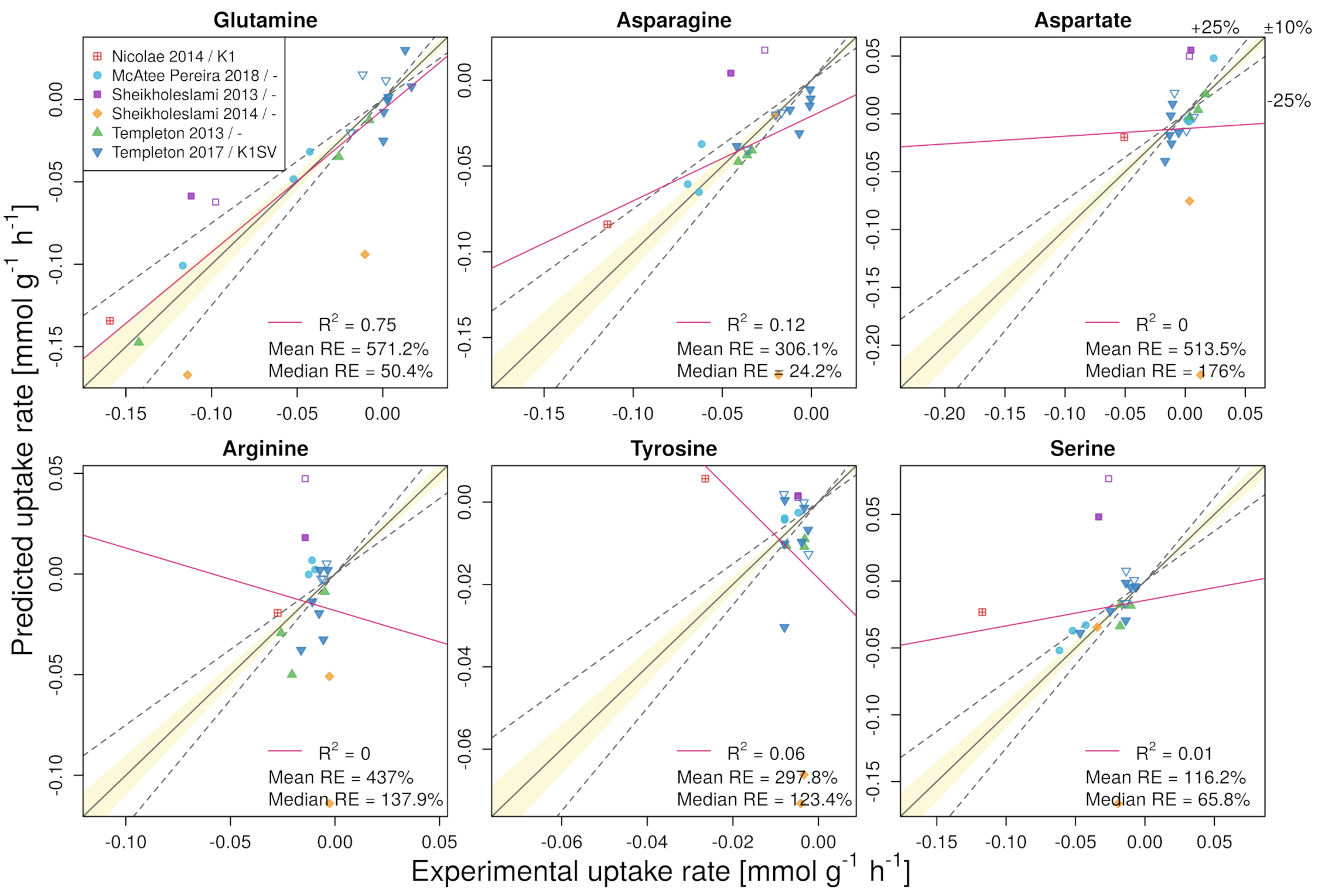

Supplement: S4 Fig — Results are shown for R_biomass_cho as the biomass reaction. RE—relative error. The legend indicates the publication and the used CHO cell line (if the information was available). Empty symbols indicate non-producers. (TIF) [file pcbi.1009022.s006.tif]

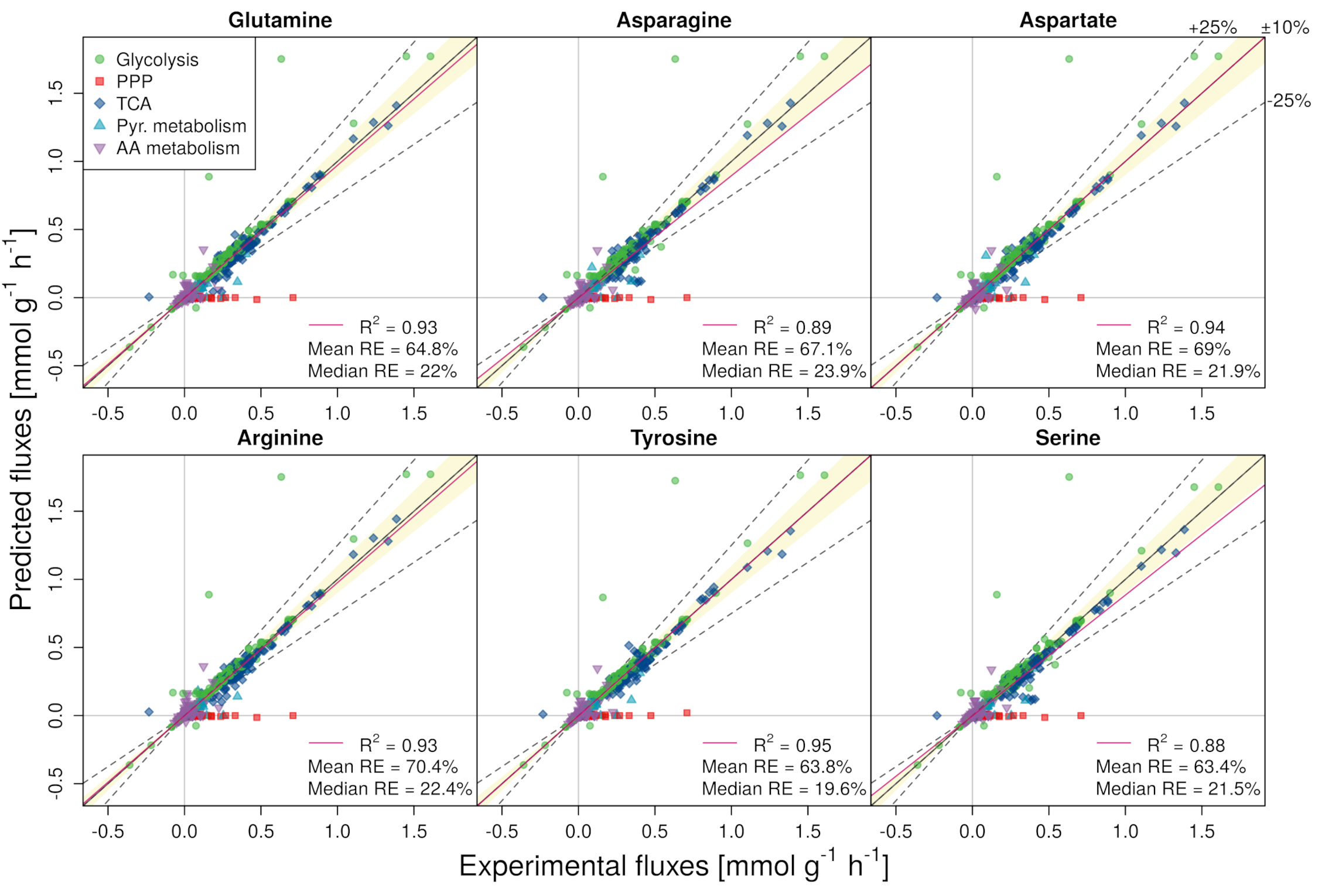

Supplement: S5 Fig — Results are shown for R_biomass_cho as the biomass reaction. RE—relative error. (TIF) [file pcbi.1009022.s007.tif]

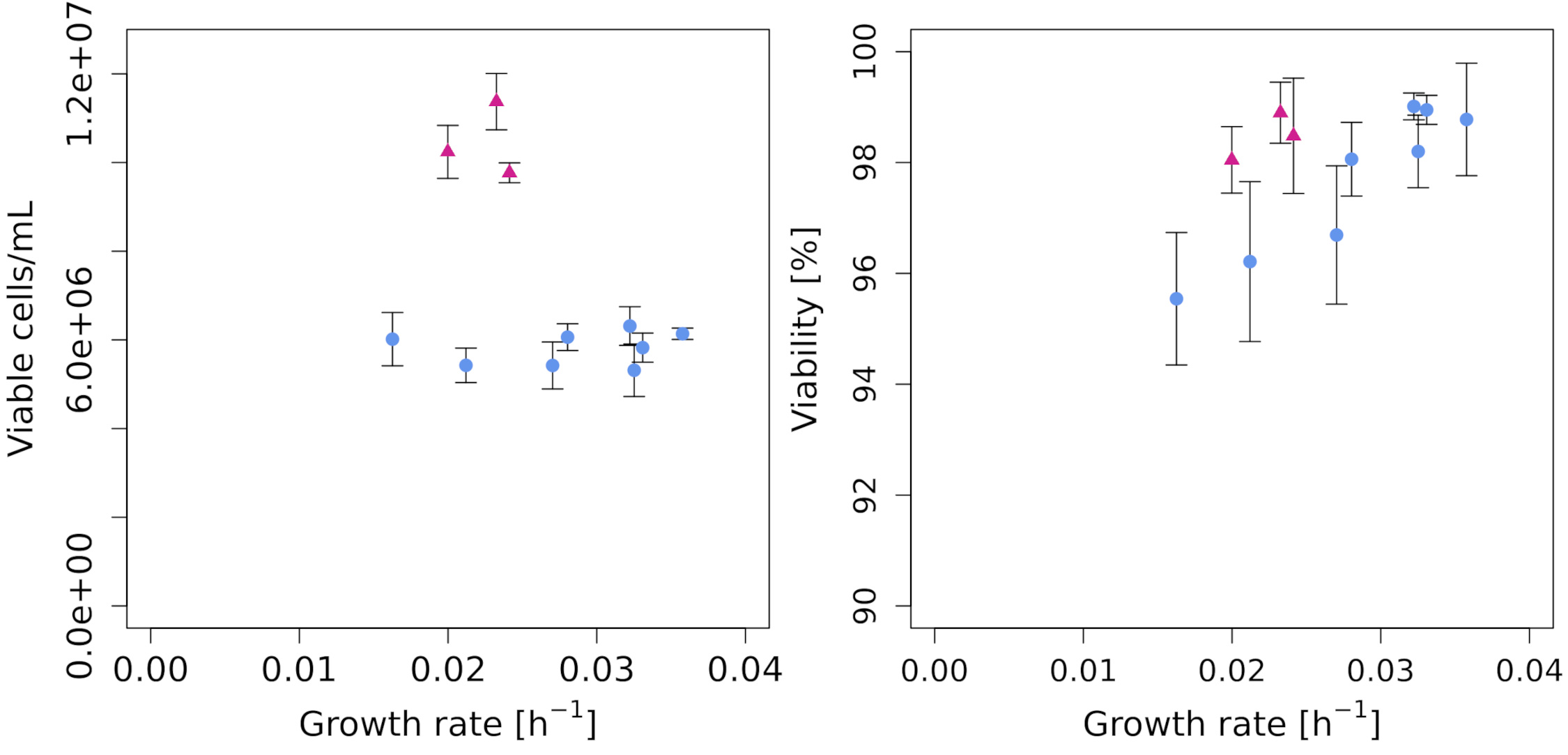

Supplement: S7 Fig — (TIF) [file pcbi.1009022.s009.tif]
