# Supplementary material for: Inclusion of maintenance energy improves the intracellular flux predictions of CHO
Source: PLoS Comput Biol. 2021 Jun 11;17(6):e1009022. doi: 10.1371/journal.pcbi.1009022 (PMC8221792; doi:10.1371/journal.pcbi.1009022)
Supplement: Attachment — Submitted filename: 113168_1_rebuttal_2025570_qqfbsv.pdf [file pcbi.1009022.s011.pdf]

Response to the comments from Reviewer #1

We would like to thank reviewer #1 for the careful evaluation of our manuscript, which we could use to significantly improve its quality. Below we address these comments individually. Comments of the reviewer are represented in blue. The shaded areas indicate additions to the manuscript.

Reviewer #1 (Evidence, reproducibility and clarity (Required)):

****Summary****

The authors examine results from and adjustments to an updated community-derived, consensus genome-scale metabolic model (GSMM) of Chinese hamster ovary (CHO) cells. The authors apply data from the literature to as well as generate new continuous culture data in order to estimate ATP maintenance energy. The report also examines the impact of using unconventional uptake rate objective functions, as well as highlight improvement in intracellular fluxes and growth rates predictions upon adding ATP maintenance energy estimates to the model.

****Major comments:****

The conclusion on alternative objectives is convincing and are consistent with those drawn in the cited article on the method.
Thank you for your encouraging overall assessment.

On point that should be clarified is that the computational method, using literature data, was reported to find that the non-producer CHO-K1 had a substantially (three times) higher ATP maintenance energy than the averages across cell lines, yet the experimental data are said to validate the method when this same CHO-K1 line has an estimated value slightly lower than the average. The data and analysis method of the experimental continuous culture seems more direct, and the authors should address this apparent issue.
Although Nicolae et al (2014) used the same (nominal) cell line as we did, we think that both data are not readily comparable. As an example, the glucose uptake in Nicolae's cell line was almost 1.7× higher than the uptake rate observed at the highest dilution rate in our data from continuous fermentation and 1.6× higher than exchange rate observed in batch cultivation of the same CHO-K1 cell line (Szeliova et al. 2020). This might be a result of either different experimental conditions (e.g. different cultivation medium and glutamine supplementation) or experimental uncertainty in the CHO-K1 literature dataset (Nicolae et al. 2014). Moreover, the exchange rates in the publication were provided in $mmol/(L_{cell}h)$ and we converted them to $mmol/(gDW\,h)$ with a dry mass value that we determined for our CHO-K1, as no dry mass was provided in the publication by Nicolae et al (2014). Both of these effects could have created a systematic shift in the exchange rates.

We addressed this issue in the discussion as follows:

The experimentally determined mATP for CHO-K1 was comparable to the average computational estimate but much lower than the estimated mATP for CHO-K1 from Nicolae et al. [20] (Fig 2a). This discrepancy might have been caused by different experimental condi-

tions, uncertainty in the exchange rates or due to conversion of the reported exchange rates from $mmol/(L_{cell}h)$ to $mmol/(gDWh)$ with a literature value for cell dry mass [13]. As an example, glucose uptake rate in Nicolae et al. was almost 1.7× higher than glucose uptake rate observed at the highest dilution rate in our continuous fermentation and 1.6× higher than the rate observed in batch cultivation of the same CHO-K1 cell line [13].

Did the authors remove fluxes that are linearly-dependent so that they are not over-counted and bias the estimations? What were the degrees of freedom in the computational estimations?
In 13C models, as many reactions as possible are typically lumped, so we did not expect that there would be any linearly-dependent fluxes (or not enough to influence the analysis). When comparing the fluxes of these lumped reactions to the fluxes in GSMM, we always calculated the net flux of the corresponding GSMM reactions to avoid over-counting.

Additional experiments and computations on other data sets are not essential to support the claims of the paper.

The data and methods are adequately presented so that they can be reproduced and adapted to other cell lines, although a table indicating the ATP maintenance energy estimates for each data set in the Supporting Information would be beneficial.
Thank you for your assessment. To support the reader we followed the reviewer's suggestion and added a table with computationally estimated mATP values for all datasets and objective functions to the supplementary material (Table S2).

In general, the statistical analysis is adequate, although the authors could comment on the improved fit that could be expected from increasing the number of fit parameters (that is, by changing the degrees of freedom), perhaps by performing χ^2 analysis.
We performed additional statistical analysis. To check whether mATP has a significant effect on the fit, we added a column to the data with mATP as a categorical variable (0 or 1) and performed linear fit with mATP as another predictor (`mATP_presence`). First we checked the effect on the slope. In an ideal case, when predicted and experimental fluxes match, the slope should be close to 1. The output below shows that the slope of the data (for `R_biomass_cho`) without mATP (column `Estimate`, row `experimental`) is 0.36 (= fluxes are underestimated). Adding mATP increases the slope by 0.62 and it is significantly different (p-value $< 2.2^{-16}$) (= the data with mATP has a slope of $0.36 + 0.62 = 0.98$, which is close to what we want.)

```
Call:
lm(formula = pFBAsum ~ experimental * mATP_presence, data = data_all,
    weights = weights, na.action = na.omit)

Coefficients:
                            Estimate Std. Error t value Pr(>|t|)
(Intercept)               -0.0007519  0.0009921  -0.758    0.449
experimental               0.3608660  0.0119206  30.273   <2e-16 ***
mATP_presence1            -0.0007140  0.0014030  -0.509    0.611
experimental:mATP_presence1  0.6165992  0.0168582  36.576   <2e-16 ***
```

We also performed χ^2 test of the models without (Model 1) or with mATP (Model 2) as a

predictor. Again, it shows that adding mATP as a predictor significantly improves the fit (p-value $< 2.2^{-16}$).

```
print(anova(fit1, fit2), test="Chisq")

Analysis of Variance Table

Model 1: pFBAsum ~ experimental
Model 2: pFBAsum ~ experimental * mATP_presence

  Res.Df    RSS Df Sum of Sq  Pr(>Chi)
1   1632 336.39
2   1630 179.82  2    156.57 < 2.2e-16 ***
```

The following paragraph was added to the Methods section:

To check whether the addition of mATP constraint has a significant effect on the fits, mATP was added as a categorical predictor (value 0 or 1) and an interaction term was included in the model (experimental fluxes*mATP). If the term is significant (p-value < 0.05), we conclude that mATP has a significant effect on the slope. We also compared the models without or with mATP predictor with χ^2 analysis.

and to the Results section:

The addition of mATP had a significant effect on the fit (p-value $< 2.2^{-16}$), leading to a significant change of the slope from 0.36 to 0.98 for `R_biomass_cho` and from 0.27 to 1 for `R_biomass_cho_producing` (a value of 1 represents a perfect agreement between experimental and predicted fluxes.)

Minor comments:

Prior studies are referenced appropriately.

The figures are clear, with clarity added through the use of symbols that differ in both color and shape.

The authors state, somewhat unequivocally, on page 11 that 13C-MFA models use small representations of metabolism. Some studies, albeit for microbial systems, have used large or near genome-scale metabolic models in conjunction with MFA. The authors could avoid this misrepresentation by inserting the qualifier "typically" or "customarily" or the like, before the word "used".
The word "typically" was added.

The authors could comment on some of the outliers in Figure S3. Are they in specific subpathways?
In most cases they belong to a specific dataset. For example in Fig S3 glycolysis +mATP, the two most overestimated points belong to the "K1" dataset. In TCA +mATP, four underestimated points again belong to "K1" dataset, one to "early" dataset. In pyruvate metabolism

+mATP, two underestimated points belong to “K1”, two to “early” datasets. In AA metabolism, the outliers belong to various datasets and sub-pathways. We added a comment to the figure legend.

Several outliers can be observed, which in most cases belong to a specific dataset. For example in glycolysis +mATP, the two most overestimated points belong to the “K1” dataset. In TCA +mATP, four underestimated points again belong to “K1” dataset, one to “early” dataset. In pyruvate metabolism +mATP, two underestimated points belong to “K1”, two to “early” datasets. In AA metabolism, the outliers belong to various datasets and sub-pathways.

Reviewer #1 (Significance (Required)):

The work signifies a technical advance in developing GSMM for CHO cells and highlights the importance of determining ATP maintenance energy for improving predictions by mammalian GSMM. The work places itself in context of recent explorations of unconventional objective functions and would be of general interest to those who apply or develop GSMM. The methods used therein could be applied to other mammalian cell lines, and the improvements described could encourage the same.
Thank you for your positive feedback.

Response to the comments from Reviewer #2

We would like to thank reviewer #2 for the careful evaluation of our manuscript, which we could use to significantly improve its quality. Below we address these comments individually. Comments of the reviewer are represented in blue. The shaded areas indicate additions to the manuscript.

Reviewer #2 (Evidence, reproducibility and clarity (Required)):

****Summary:****

This manuscript illustrates the authors' improvement of pFBA predictions of growth rates and most intracellular fluxes (except fluxes in pentose phosphate pathway) for producing- and non-producing CHO cells. The authors utilized previously reported 13C experimental metabolic flux datasets to validate intracellular flux predictions under both maximized biomass and minimized nonessential metabolites uptake simulation objectives. The authors demonstrated significant improvement of predictions by incorporating maintenance energy constraints into the genome scale metabolic model. The improvement of predictions on CHO intracellular fluxes and the computational estimation of CHO maintenance energy, which was well comparable to the maintenance energy experimentally determined by the authors, had not been reported by others.

****Detailed Comments:****

This manuscript is concise and logically meticulous with full explanations or discussions on methods and discovered phenomena to prove the idea that incorporation of maintenance energy in the genome scale metabolic model can improve the accuracy in predicting not only growth rates, which had been accurately predicted in previous reports, but also intracellular fluxes in CHO cell lines. This manuscript can be accepted if below minor problems can be further explained or fixed:
Thank for your positive evaluation.

1. The authors found that predictions on CHO growth rate is less accurate without adding the maintenance energy constraint, but according to the introduction (line 24), researchers had achieved ACCURATE prediction of CHO growth rates without applying maintenance energy constraints. What is the difference between these growth rate predictions?

We added the following paragraph to the discussion:

In our previous contribution we observed that if glucose uptake and *all* amino acid exchange rates are accurately measured, growth rate predictions are accurate too as these rates essentially determine the energy metabolism even without considering mATP. Here we encountered several data sets where the inclusion of mATP improved growth predictions. However, since accuracy estimates on the exchange rates were sometimes missing in the original publications, we are unable to exclude simple measurement inaccuracies as the reason for this observation.

2. Except predictions on glucose uptake, predictions on metabolite uptakes were not accurate under the minimized uptake simulation objective even with incorporation of the maintenance, and there seems to be not much explanation in the discussion section. Is there any possible detailed explanation on it?

We added a possible explanation to the discussion:

A possible explanation could be that the uptake rates of glucose are higher than the amino acid uptake rates, so the relative error is smaller and the predictions are less influenced by the noise in the input data.

3. In Table 1, 'steady state concentrations of metabolites' are not clear. Are they exchange rates? If not, what are their definitions? How were they calculated?

Thank you for pointing out this mistake in the table caption. The table indeed shows metabolite exchange rates, calculated with Eq. 4. The caption was corrected and reference to the equation used for the calculation was added:

The dilution rates, calculated growth rates (Eq(3)), steady state concentrations of cells, metabolite exchange rates (Eq(4)) and carbon recovery.

4. What fluxes are used for experimental estimation of mATP? Are they the fluxes of metabolites described in the manuscript? Please expand on the "Determination of Maintenance Energy" section within Materials and Methods.

We slightly changed the calculation method as a response to the comments from reviewer #3. We provided more details in the Methods section as follows:

After constraining the growth rate and extracellular exchange rates to the experimental values, pFBA was performed with maximization of ATP hydrolysis as the objective (reaction `R_DM_atp_`) for each dilution rate. Total ATP production was calculated by summing up fluxes of all reactions in the GSMM that produce nucleoside triphosphates (ATP, GTP, CTP, UTP, TTP, ITP, dATP, dGTP, dCTP, dUTP, dTTP, dITP; these compounds can be interconverted in the model) and plotted against growth rates. Linear fit was done in R (function `lm`). The intercept represents the estimated non-growth associated energy consumption (mATP) and 1/slope is biomass yield per mole of ATP corrected for mATP (Y_{ATP}^{max}).

5. What is the difference between R_biomass_cho and R biomass cho producing? Please add a little description on it.

We added the following sentence explaining the main differences between the biomass functions to the Methods section:

Maximization of biomass production was used as the objective function using two biomass reactions available in the GSMM, `R_biomass_cho` and `R_biomass_cho_producing`. The main difference between these two reactions is that `R_biomass_cho` has lower protein content (56% compared to 70% in `R_biomass_cho_producing`), but higher lipid, DNA and RNA content.

6. What is the purpose of Figure 5? Can it be put in the supplementary information?
The figure shows the uptake rates of the major energy sources and secretion rates of the main waste products. We wanted to illustrate how they change at the different dilution rates. Glucose, glutamine and lactate exchange rates are key inputs for the calculation of the maintenance energy. Furthermore, they are often the main parameters determined for CHO cell cultures, so they could be interesting for other scientists working with CHO (also in other areas of CHO research). That is why we'd prefer to keep Figure 5.

7. In line 268, there is a term 'Enforcing a minimal mATP'. What does it mean? Does it mean 'adding estimated mATP value as the constraint'? How does 'minimal' mATP constraint compare to estimated average mATP?
Yes, it means adding the mATP as constraint. We rewrote the sentence to avoid the ambiguity:

Adding the estimated mATP value as a constraint strongly decreases the prediction errors in the intracellular fluxes for all but one dataset, see Fig 2b.

8. Please correct R2 in the right part of table 2 as superscript.
Thank you, the table was corrected.

9. How do the different cell lines and cultivation conditions of the 13C MFA datasets affect the prediction accuracies and ability to estimate mATP? Please expand on this in the discussion of line 347.
The differences between the studies do not involve only cultivation conditions/cell lines, but also different labelling approaches (detection of extracellular vs. intracellular metabolites, different types of labelled substrates), quantification methods and modelling approaches (steady state vs. non-stationary modelling, presence of compartments), so it is difficult to separate the individual effects on the estimation of mATP. We added the following paragraph to the discussion:

However, it was not possible to differentiate between biological effects on mATP estimation (e.g. cell lines, conditions) from the effects caused by the differences in labelling and quantification (different labelled substrates, detection of intracellular vs. extracellular labelled metabolites) and modelling approaches (e.g. steady state vs. non-stationary modelling, presence of compartmentalisation).

Reviewer #2 (Significance (Required)):

The authors provide a valuable contribution to the metabolic modeling efforts of mammalian systems. By improving the accuracy of intracellular flux predictions of CHO cells, future metabolic engineering efforts in CHO cells can be more appropriately focused providing significant value in both time and money. Additionally, the authors help to bridge the gap between theory and application by using existing 13C MFA datasets to validate their efforts. Furthermore, the authors provide further evidence for the validity of incorporating maintenance energy constraints which future researchers can apply in other systems. Ultimately, these achievements could benefit CHO cell simulations, which would help shorten CHO cell

line development biopharmaceutical productions. Though we have limited experience with mammalian systems, we have significant expertise with metabolic engineering of bacterial systems.

Thank you for your positive feedback.

Response to the comments from Reviewer #3

We would like to thank reviewer #3 for the careful evaluation of our manuscript, which we could use to significantly improve its quality. Below we address these comments individually. Comments of the reviewer are represented in blue. The shaded areas indicate additions to the manuscript.

Reviewer #3 (Evidence, reproducibility and clarity (Required)):

Szeliova and colleagues use a Genome-Scale Metabolic Model (GSMM) of CHO cells to evaluate the ability/validity of this modeling to determine the intracellular fluxes of central metabolism from these cells under chemostat culture conditions. Using metabolic flux determinations using 13C data from a wide range of conditions that include batch, fed-batch and semicontinuous cultures, they found that this modeling approach is unable to accurately describe the intracellular fluxes of CHO cells through major pathways of central catabolism. They perform their own chemostat cultures of CHO cells at different dilution (growth) rates and estimate the non-growth associated energy maintenance in ATP. When they include this constraint in the GSMM model they are able to estimate fluxes more accurately.

There are several questions/issues that this Reviewer has for this work which need to be addressed.

1. Which is the respiratory quotient (RQ) of their own CHO cells culture reported in Table 1? Without knowing RQ is very difficult to assess the reliability of their flux estimation because of the lack of knowledge about the extent of mitochondrial oxidative phosphorylation, thus their estimated fluxes through the tricarboxylic acid (TCA) cycle.
Unfortunately, we were not able to measure O_2 consumption in our experimental set up. So we cannot calculate the RQ. Because of that, we estimated ATP consumption with the use of the CHO GSMM based on the measured growth rate and consumption and secretion rates of glucose, glutamine, lactate and 19 other extracellular metabolites.

2. How do they know whether the CHO cells are consuming the extracellular lactate released? Specifically, in their Table 1, DRs 8, 10 and 11, where the steady state values of lactate are lower compared to the other DRs that look reasonably similar within experimental error (excepting DR3 due to contamination). Coincidentally, the steady state values of glucose for the DRs mentioned were lower, and it is known that CHO cells will consume lactate happily, thus producing a mixed substrate culture, which will change pathways utilized by CHO, notably mitochondrial metabolism because of lactate conversion into pyruvate before being metabolized by mitochondria. Incidentally, it is known that when CHO cells consume glucose preferentially, they proliferate faster than when using lactate as substrate, as can be appreciated by the corresponding decrease in growth rate reported in Table 1, that correspond to changes in doubling time of the cell culture from 31 h (average =0.022 for DRs: 8, 10, 11) to 22 h (average =0.032 for DRs: 5-7). All this should be clarified/discussed.
We analysed the cultures in steady states, which means the consumption/secretion rates were constant. As the incoming medium does not contain any lactate, but there is lactate in the outgoing medium, we know that lactate has to be produced (according to Eq. (4)). Lactate may have been consumed in the transition periods between the different steady states, but we do not have the data to verify this as we were only focused on the steady state analysis.

It is also possible that the transition from one steady state to another could have changed

the metabolic state of the cells (multiple steady states can exist at the same conditions, see references in the discussion). At the lower rates (DR8, 10 and 11) the metabolism could have switched to a more efficient metabolic state with more active TCA cycle and oxidative phosphorylation. This would explain the different metabolic profiles of these three dilution rates. We briefly touched on this issue in the first version of the manuscript in lines 395-397, but we expanded the discussion to bring more attention to it:

Furthermore, the physiological state of a culture during steady state might differ depending on how it was reached and different properties (e.g. cell and metabolite concentration) can be observed even if the same dilution rate and cultivation conditions are used [40–47]. Such multiplicity of steady states is likely a consequence of toxic waste product accumulation. Lower waste product secretion and higher cell densities indicate a metabolic switch to an energetically more efficient metabolism (higher activity of TCA and oxidative phosphorylation). This phenomenon could explain the different cell densities and exchange rates observed for three dilution rates (DR8, DR10 and DR11; Fig S7 and Fig 5). During the transitions between different steady states, cells could have switched to a more oxidative metabolism with lower lactate secretion (or even consumption during the periods of transition). However, not enough data was available to investigate this phenomenon in more detail.

3. Since cancer cells like CHO display aerobic glycolysis (Warburg effect) thus a lot of C is lost for the biomass via lactate release to the extracellular medium. What is the C-recovery of their cultures reported in Table 1? This should be reported because without knowing if the carbon is recovered quantitatively, the Authors would be blind to the potential activation of other metabolic pathways than those they are considering, which could be gleaned from the exometabolome in the culture medium.
We calculated C-recovery from the metabolite exchange rates, growth rate and biomass composition from Szeliova et al. 2020. For 10/11 cultures C-recovery was at least 85%, which suggests that we quantified the most important secretion rates. For the lowest dilution rate (DR3), it was 70%. Compared to the dilution rate with the next closest growth rate (DR10), it had similar total C-secretion, but higher C-consumption. Possible explanations include experimental error or that the culture did not reach a steady state. The calculated C-recovery values were added to Table 1.

4. Experimentally, since the Authors have a range of dilution rates (D), and in a plot of specific rate of ATP production, qATP, vs. D qATP $= 1/(Y_{ATP}^{max})$ D + mATP they can determine energy maintenance in ATP, mATP, this Reviewer is curious about what kind of expression for calculating qATP they would use for the cultures reported in Table 1, and what value of the growth yield per mole of ATP, Y_{ATP}^{max} (corrected for energy of maintenance), they obtain from the slope of such a plot, and how that value compares with their own, and others reported in the literature for cancer cells or rapidly proliferating cells?
Table 1 shows results from our continuous cultivations which were used to calculate qATP values in Figure 6. To make it more clear, the figure caption was changed to

The rate of ATP consumption at different growth rates (as indicated in Table 1).

The original version Figure 6 could not be used to calculate Y_{ATP}^{max} because it did not show the total ATP consumption, but the maximum flux of ATP hydrolysis reaction while constraining growth – it does not include the energy used for the biomass synthesis in the model.

Therefore, we changed our approach and calculated total ATP production by summing up all reactions that produce ATP (or other nucleoside triphosphates). The method description was changed as follows:

After constraining the growth rate and extracellular exchange rates to the experimental values, pFBA was performed with maximization of ATP hydrolysis as the objective (reaction `R_DM_atp_`) for each dilution rate. Total ATP production was calculated by summing up fluxes of all reactions in the GSMM that produce nucleoside triphosphates (ATP, GTP, CTP, UTP, TTP, ITP, dATP, dGTP, dCTP, dUTP, dTTP, dITP; these compounds can be interconverted in the model) and plotted against growth rates. Linear fit was done in R (function `lm`). The intercept represents the estimated non-growth associated energy consumption (mATP) and 1/slope is biomass yield per mole of ATP corrected for mATP (Y_{ATP}^{max})).

Maintenance energy calculated with this method was almost the same as previously (4.3 ± 1.7 vs. 4 ± 1.6). The calculated Y_{ATP}^{max} was 5.7 ± 2.1 g/mol ATP.

We added following text to the results section:

From the slope of Fig 6 we also calculated Y_{ATP}^{max} (growth yield per mole of ATP corrected for maintenance energy) and obtained value of $5.7 \pm 2.1\ \mathrm{g\,mol^{-1}}$.

And to the discussion:

The estimated Y_{ATP}^{max} is close to previously reported values in yeast where it ranges from $8.6\ \mathrm{g\,mol^{-1}}$ [58] up to 9.5 or $25.1\ \mathrm{g\,mol^{-1}}$ depending on the carbon source [59]. Values for bacteria are also in a wide range between 10 to $31.9\ \mathrm{g\,mol^{-1}}$, also depending on the carbon source [60]. To our knowledge, this information is not available for mammalian species up till now.

Moreover, if they have evidence that the CHO cells in their cultures are using oxidative phosphorylation, how sensitive would be their qATP determinations to the P/O ratio?

iCHO1766 uses P/O ratio (NADH) of 2.5, which is generally the standard value. We varied the ratio between 2-3 to test the effect on mATP estimation with our experimental data. We added Figure S8 and a following paragraph to the Results section:

Because energy is partially generated via oxidative phosphorylation, the amount of produced ATP depends on the P/O ratio. In *i*CHO1766, P/O ratio for NADH is 2.5, which is a standard value [33]. To check how this value affects mATP estimation, we varied P/O ratio between 2-3 and obtained mATP values between 3.5-5 $\mathrm{mmol\,g^{-1}\,h^{-1}}$ (Fig S8).

5. On the basis of their worse flux prediction of the pentose phosphate pathway (PPP) after accounting for the energy of maintenance in ATP, they point out "a possible lack of actual NADPH demand in the model, e.g., for protein folding or degradation of misfolded proteins". However, the Authors omit to mention the crucial anabolic requirements of NADPH, which will rely mainly on the PPP. In the context of the Authors' work, this omission is not acceptable with rapidly proliferating cells. Other NADPH sources would be the mitochondrial

transhydrogenase, present in all mammalian cells (not in yeast), and the NADP-dependent isocitrate dehydrogenase.
We added additional clarification to the discussion, which points out the anabolic demands for the synthesis of lipids and nucleotides.

The 13C data contains activities of malic enyzme (ME) and isocitrate dehyrogenase (ICDH) (as a part of lumped reaction from citrate to alphaketoglutarate), but it does not distinguish between the NADH and NADPH producing enzymes, so we cannot verify the accuracy of the predictions for the individual enzymes, only the sum of the fluxes. We checked how the activities of the different enzyme versions changed after the addition of mATP constraints, but no consistent changes were observed.

We expanded the discussion as follows:

Fluxes of PPP got worse after the addition of a mATP constraint, which points at alternative NADPH sources connected to the TCA, e.g. $NADP^+$-dependent malic enzyme (NADP-ME) or $NADP^+$-dependent isocitrate dehydrogenase (NADP-ICDH). Indeed we found higher activity of NADP-ME and NADP-ICDH in some datasets, but not consistently in all. This points to a possible lack of actual NADPH demand in the model. Anabolic pathways that require NADPH, such as synthesis of lipids or nucleotides [35] are present in the model. However, additional NADPH-consuming processes such as protein folding, degradation of misfolded proteins [36] or maintenance of cellular redox balance [37] are not represented in the model.

Reviewer #3 (Significance (Required)):

Although this Reviewer would agree with the proposal that the addition of the ATP expense in non-growth associated maintenance (e.g., transport, polymerization, molecular assembly and organization) will contribute to reduce while circumscribing the volume of possible solutions thus decreasing the flux variability that they report in the manuscript, there is a more trivial explanation which is that they use data (20 datasets) proceeding from culture conditions that do not comply with the steady state condition required to apply GSMM modeling rigorously. Since this is a more trivial explanation of the broad flux determination error, they should evaluate comparatively their potential relative impact on the work's conclusions.

It is important to clarify that GSMM modeling produces a space (volume) of possible solutions, within which some of those solutions will correspond to the intracellular fluxes that the Authors describe. This could also explain the broad variability of their flux estimation. It would be important to state this clearly mainly for the reader non-expert on this topic.
The literature datasets cover a range of different culture modes, including semi-continuous, batch (including early, late and stationary phases) and fed-batch cultivations, some of which are commonly considered to be in pseudo-steady state and used for modelling with FBA (with successful results). Furthermore, despite the variability in the culture conditions, the addition of mATP consistently improved the predictions of intracellular fluxes. Moreover, we used pFBA, which minimized the sum of fluxes and therefore drastically reduces the solution space.

We expanded the discussion to clarify this issue:

Finally, it is important to note that GSMMs have a large solution space. By computing a parsimonious flux distribution the space of possible solutions is reduced. That space can be further constrained by imposing mATP demand. However, some variability will remain (which may partially explain the variability in the mATP estimates). Hence, adding even more constraints could be beneficial for model performance. As an example, Lularevic et al. [55] reduced variability in flux variability analysis of *i*CHO1766 by adding carbon availability constraints. In another study, the predictions of intracellular fluxes were improved by adding constraints based on enzyme kinetic information [9]...
