# Supplementary material for: Inclusion of maintenance energy improves the intracellular flux predictions of CHO
Source: PLoS Comput Biol. 2021 Jun 11;17(6):e1009022. doi: 10.1371/journal.pcbi.1009022 (PMC8221792; doi:10.1371/journal.pcbi.1009022)
Supplement: Peer review history

# Author comment: Author response to previous submission

**DOI:** 10.1371/journal.pcbi.1009022.r001

14 Apr 2021

Attachment Submitted filename: 113168_1_rebuttal_2025570_qqfbsv.pdf Click here for additional data file.

# Editor report: Decision Letter 0

**DOI:** 10.1371/journal.pcbi.1009022.r002

**Authors:** Jason M. Haugh

28 Apr 2021

Dear Mr. Ruckerbauer,

We are pleased to inform you that your manuscript 'Inclusion of maintenance energy improves the intracellular flux predictions of CHO' has been provisionally accepted for publication in PLOS Computational Biology.

Before your manuscript can be formally accepted you will need to complete some formatting changes, which you will receive in a follow up email. A member of our team will be in touch with a set of requests.

Please note that your manuscript will not be scheduled for publication until you have made the required changes, so a swift response is appreciated.

IMPORTANT: The editorial review process is now complete. PLOS will only permit corrections to spelling, formatting or significant scientific errors from this point onwards. Requests for major changes, or any which affect the scientific understanding of your work, will cause delays to the publication date of your manuscript.

Should you, your institution's press office or the journal office choose to press release your paper, you will automatically be opted out of early publication. We ask that you notify us now if you or your institution is planning to press release the article. All press must be co-ordinated with PLOS.

Thank you again for supporting Open Access publishing; we are looking forward to publishing your work in PLOS Computational Biology. 

Best regards,

Jason M. Haugh

Deputy Editor

PLOS Computational Biology

***********************************************************

# Editor report: Acceptance letter

**DOI:** 10.1371/journal.pcbi.1009022.r003

**Authors:** Jason M. Haugh

1 Jun 2021

PCOMPBIOL-D-21-00677 

Inclusion of maintenance energy improves the intracellular flux predictions of CHO

Dear Dr Ruckerbauer,

I am pleased to inform you that your manuscript has been formally accepted for publication in PLOS Computational Biology. Your manuscript is now with our production department and you will be notified of the publication date in due course.

The corresponding author will soon be receiving a typeset proof for review, to ensure errors have not been introduced during production. Please review the PDF proof of your manuscript carefully, as this is the last chance to correct any errors. Please note that major changes, or those which affect the scientific understanding of the work, will likely cause delays to the publication date of your manuscript. 

Soon after your final files are uploaded, unless you have opted out, the early version of your manuscript will be published online. The date of the early version will be your article's publication date. The final article will be published to the same URL, and all versions of the paper will be accessible to readers.

Thank you again for supporting PLOS Computational Biology and open-access publishing. We are looking forward to publishing your work! 

With kind regards,

Katalin Szabo

PLOS Computational Biology | Carlyle House, Carlyle Road, Cambridge CB4 3DN | United Kingdom ploscompbiol@plos.org | Phone +44 (0) 1223-442824 | ploscompbiol.org | @PLOSCompBiol
